# The effects of physical emotion intensity and perceived emotions on trait and ability judgments are non-linear

**DOI:** 10.1038/s41598-026-65262-5

**Published:** 2026-08-17

**Authors:** Heidi Mauersberger, Shlomo Hareli, Ursula Hess

**Affiliations:** 1 Department of Psychology, Humboldt-Universität zu Berlin, 12489 Berlin, Germany; 2 School of Business Administration, University of Haifa, 3498838 Haifa, Israel

**Keywords:** Blended emotions, Facial expression perception, Social judgment, Trait attribution, Expresser gender, Non-linear modeling, Neuroscience, Psychology, Psychology

## Abstract

Facial expressions are important cues for trait inferences, yet most research focuses on intense, prototypical displays, leaving open how observers interpret ambiguous or blended expressions. Across two studies, we examined how anger–sadness morphs shape emotion perception and social judgments, and whether these effects differ by expresser gender. Study 1 revealed categorical perception of anger–sadness blends, with male faces categorized as angry at lower physical anger intensities than female faces. Study 2 showed that female faces were perceived as sadder and male faces as angrier primarily at lower physical anger intensities. Social judgments varied non-linearly with perceived anger and sadness, with the pattern differing by judgment domain and expresser gender. Higher perceived anger predicted greater dominance, lower affiliation, and, for male expressers, greater leadership ability, whereas higher perceived sadness predicted lower dominance and leadership ability. Crucially, gender differences in social judgments were reduced once perceived anger and sadness were taken into account. Thus, anger–sadness morphs reveal socially structured, non-linear patterns of emotion perception and person judgment that may be missed by focusing only on prototypical endpoint expressions.

## Introduction

People rapidly and spontaneously make judgments about the personality of others based on appearance cues (see e.g.^1–3^). Next to such static features of the face, facial expressions are potent cues used by observers to attribute traits and abilities to others. Emotion expressions such as anger and sadness, among others, are linked to attributions of dominance and affiliation^4,5^.

However, much of this research has focused on relatively intense, prototypical facial expressions. Yet such stimuli are quite different from emotional expressions in naturalistic social interactions, where individuals rarely display full-face prototypes^6,7^. In fact, everyday emotional displays tend to be ambiguous, fractional, or blended. Critically, such expressions should not be treated as weaker or noisier versions of pure expressions; rather, these ambiguous signals may engage partly different perceptual and inferential processes. Consistent with this view, recent work has emphasized that people frequently both experience and display mixed emotions^8,9^ highlighting the need to assess emotion recognition beyond single, prototypical expressions^10^. This raises the question of how such ambiguous or mixed emotional expressions shape person perception, particularly as observers use facial expressions to infer traits and abilities^11,12^. Hence, the goal of the present research was to assess the effect of blended emotion expressions on perceived traits and abilities, taking into account expresser gender. Notably, as gender also affects perceived emotion intensity for a given expression shown by a man or a woman^13^ trait judgments can be due to either the physical intensity of the expression, the perceived intensity of the expression or gender stereotype judgments independent of perceived or physical expression intensity. Disentangling these effects for blended emotions was the goal of the present study.

### Perceiving traits and abilities from facial expressions

Emotion expressions can inform observers about a person’s character by a process called reverse engineering or reverse appraisal^11,12^. Specifically, according to appraisal theories of emotion, a relevant change in the environment is appraised along several dimensions and emotions are differentiated by the pattern of appraisals that give rise to them. Thus, anger is characterized by appraisals of goal obstruction, high coping potential and norm violation. Conversely, sadness is characterized by appraisals of goal obstruction combined with low coping potential^14^. Importantly, how a given situation is appraised is specific to the individual and the individual’s current state. Factors such as the personality and skills of the person determine their resources, values and motivations. These in turn define the outcome of their appraisal of an event.

Notably, people can reconstruct the appraisals that underlie other people’s emotions (e.g.^15–17^). This, in turn, provides insights into their goals, values and motivations and through these into their character^11,12^. For example, if someone reacts with anger to an injustice, an observer may infer that the person has values according to which the event in question appears unjust, perceives this injustice as incongruent with their desire to see justice done and feels competent to act accordingly. Since this process relies on the appraisal attribution of the observer, stereotype-based knowledge about the expresser enters this process. Thus, gender-based stereotypes and normative expectations about men’s and women’s emotional reactions can lead to differential appraisal attributions and consequently differential attributions of traits and abilities.

### Stereotype effects

Traditionally, gender stereotypes describe men as more agentic and women as more communal (e.g.^18^). More recent work confirms this general stereotype but also points to the importance of considering intersectionality, for example with age, which can qualify and even reverse this pattern^19^. Yet, in the absence of such factors, we would expect women to be perceived as more affiliative and men as more dominant and in control. Gender stereotypes regarding emotional expressivity suggest that men are expected to show more anger, whereas women are expected to show more sadness^20,21^. In turn, anger implies relatively high coping potential and agency, whereas sadness implies low coping potential^14^. Thus, gendered expectations about who is likely to show anger or sadness may influence the interpretation of a given expression in terms of perceived intensity and social judgment.

### Blended emotions

As noted above, the research discussed so far is based on expressers showing relatively intense prototypical expressions. An exception is a study by Hess et al.^4^ who investigated the effects of expresser gender and ethnicity on trait perceptions from emotion expressions at 40% and 80% intensity. Hess et al.^4^ proposed that trait attributions from emotional expressions depended on both perceived emotion and stereotype-based expectations regarding how likely a given expresser is to show that emotion, based on expresser characteristics such as gender and ethnicity. Importantly, their findings showed that expression intensity did not affect dominance and affiliation judgments uniformly: affiliation ratings were influenced by both low- and high-intensity expressions, whereas dominance ratings were affected mainly by more intense expressions. However, Hess et al.^4^ did not study emotion blends.

This was done by Kaminska et al.^22^ who presented observers with blended expressions morphed from 100% anger to 100% happiness and found that when expressers showed ambiguous expressions on this continuum they were rated as less trustworthy. However, because participants did not rate perceived emotion intensity, it remained unclear whether the observed social judgments were driven by the physical morph level itself or by the emotion intensity perceived by observers.

Notably, work on categorical perception of facial expressions shows that gradual physical changes along an expression continuum can produce non-linear changes in perceived emotion, especially around category boundaries^23,24^. Specifically, ratings often follow an S-shaped curve: changes at the extremes of the continuum have relatively little impact, whereas small changes in the midrange can strongly shift perception—indicating that emotion perception is categorical. In a morph continuum, physical intensity refers to the objective proportion of one endpoint expression in the stimulus, whereas perceived intensity reflects the observer’s subjective interpretation of the expression.

To the degree that the perception of traits and abilities is driven by perceived emotional intensity, a non-linear effect would be expected for their attribution as well. However, the proposal by Hess et al.^4^ points to the importance of expresser gender, which may moderate the relationship between physical emotion intensity, perceived emotion intensity and the attribution of traits and abilities.

## The present research

We conducted two studies to examine how physical anger intensity along an anger–sadness continuum is transformed into perceived emotion intensity and how perceived emotions, in turn, predict trait and ability judgments. Study 1 tested whether anger–sadness blends are perceived categorically and whether the category threshold differs by expresser gender. Based on gendered emotion expectations^20,21^ or facial morphology effects that make anger more salient when shown by men^25–27^, we predicted that anger would be categorized at lower physical anger intensities in male than in female faces.

Study 2 examined whether physical anger intensity predicted perceived anger and sadness non-linearly, whether these functions differed by expresser gender and whether trait and ability judgments were better explained by perceived emotion intensity than by physical intensity alone. Based on reverse-appraisal accounts of trait inference from emotion expressions^11,12^ and previous findings linking anger and sadness to dominance and affiliation judgments^4,5^, we expected perceived anger to increase perceived dominance and reduce perceived affiliation, whereas perceived sadness was expected to reduce perceived dominance and leadership ability. We further expected that gender differences in trait and ability judgments would be reduced once perceived anger and sadness were included in the models.

## Study 1

**Fig. 1:**
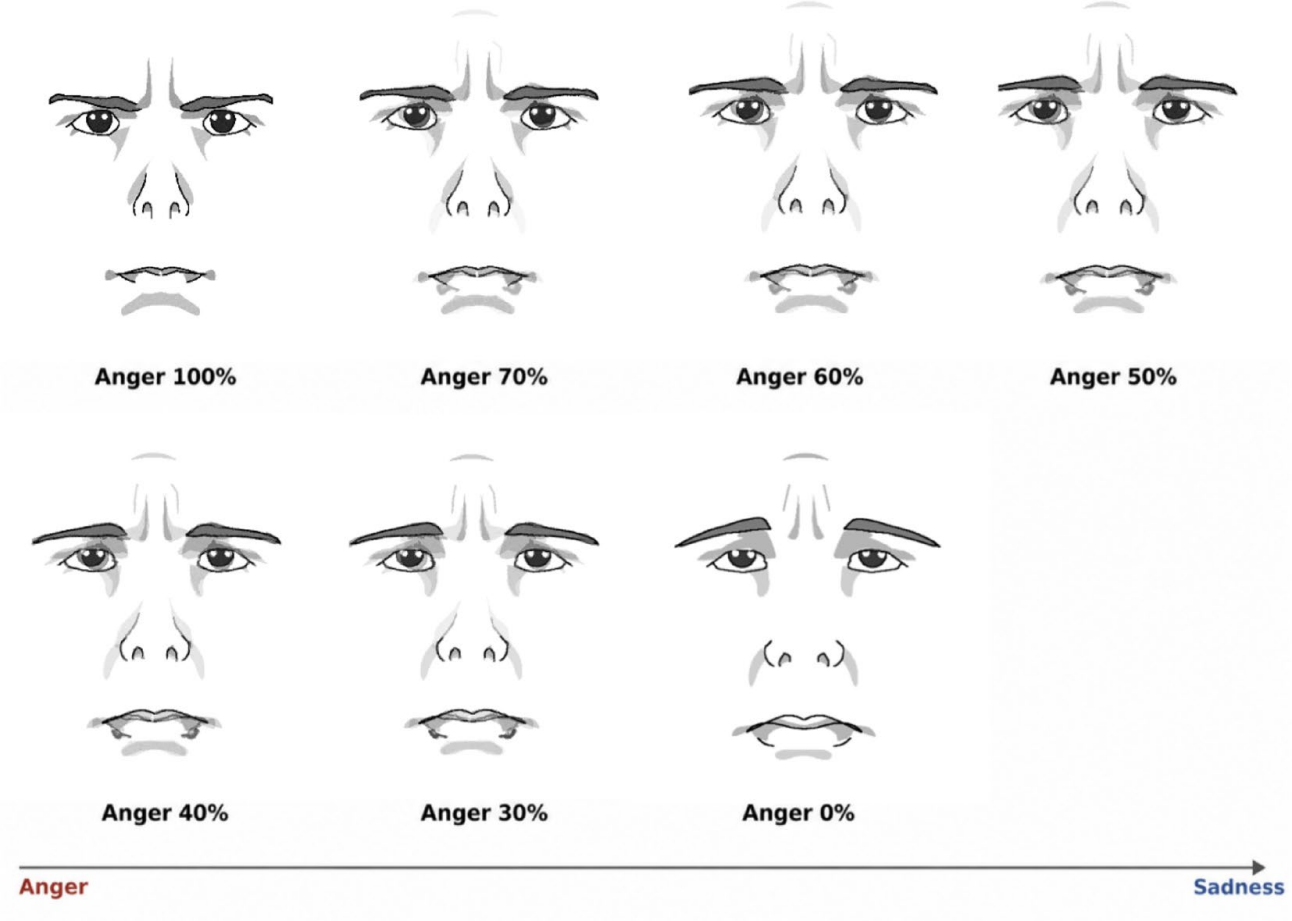
Illustrative male anger–sadness morph continuum based on morphed hand-drawn faces from Ursula Hess’s archive. The full continuum varied in 10% steps from 100% physical anger intensity (0% sadness) to 0% physical anger intensity (100% sadness). The original study stimuli were static, color facial morphs derived from a limited set of identities from the young-adult subset of the FACES database^28^. Because these photographs depicted real, identifiable individuals and could not be reproduced under the applicable copyright restrictions and Springer Nature’s licensing requirements, the present drawings are shown to illustrate the continuum.

Participants saw either 110 male or 110 female faces with emotions blended from 100% Sadness to 100% Anger in 11 steps (see Fig. 1). In a speeded forced-choice task, they categorized each expression as either sad or angry.

### Results

#### Categorical ratings

We analyzed the categorization of facial expressions as either sad or angry via a generalized linear mixed-effects model (GLMM) with a binomial link function using lme4^29^ in R^30^. Physical anger intensity and expresser gender were entered as fixed effects with random intercepts for participant and random slopes for physical anger intensity. Parameters were estimated using Laplace approximation and the bobyqa optimizer.

**Fig. 2:**
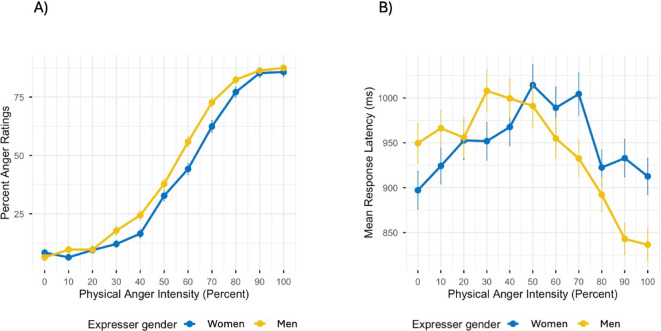
(**A**) Categorical ratings and (**B**) response latencies as a function of physical anger intensity and expresser gender. A value of 0% physical anger corresponds to 100% sadness, whereas 100% physical anger corresponds to 0% sadness.

As expected, a significant main effect of physical anger intensity, ß = 6.37, CI_95%_ = [5.97, 6.77] z = 31.26, *p* < 0.001, emerged. The relationship between anger ratings and physical anger intensity followed a sigmoid curve, indicating categorical perception (see Fig. 2A).

Further, a significant main effect of expresser gender, β = − 0.17, CI_95%_ [− 0.30, − 0.04], z = − 2.61, *p* = 0.009, emerged, such that male expressers were categorized as angry at lower physical anger intensities. Specifically, the point of subjective equality (PSE), derived from the fitted mixed-effects model, occurred at 53.13% physical anger for male expressers, compared with 57.37% for female expressers. The interaction was not significant, β = 0.04, CI_95%_ [− 0.15, 0.23], z = 0.41, *p* = 0.682.

#### Response latency

An ANOVA type LMM with the factors physical anger intensity and expresser gender as well as random intercepts for participant on the latency ratings yielded a significant main effect of physical anger intensity, F(10, 9114) = 14.80, *p* < 0.001, η^2^_p_ = 0.02 and a significant interaction, F(10, 9114) = 5.42, *p* < 0.001, η^2^_p_ = 0.01. The main effect of expresser gender just failed to be significant, F(1, 9114) = 3.77, *p* = 0.052, η^2^_p_ = 0.00. Latencies were longer in the midrange of the physical intensity continuum, consistent with greater categorization difficulty when neither emotion clearly dominated (see Fig. 2B). In line with the notion that anger is easier to decode in male faces, response latencies became shorter for male expressers as anger intensity increased. At low anger intensities, i.e., high sadness intensities, response times were faster for female expressers (for more detail see supplement available at OSF).

### Discussion

As expected, blended angry-sad expressions were perceived categorically; whereas increases in physical anger intensity had very little effect at the two extremes of the continuum, small changes had a substantial effect in the midrange. Further, response latencies suggested that labeling the expressions was easier for blends where one emotion clearly dominated. Importantly, as predicted, anger was categorized at lower physical anger intensities in male than in female faces. Response latencies were broadly consistent with this pattern, suggesting lower categorization difficulty for male expressers as anger intensity increased. Conversely, participants responded faster for female expressions that were high in sadness. Crucially, the model-based category threshold (PSE) was lower for male than for female expressers. Based on these results, we investigated the attribution of traits and abilities to expressers as a function of emotion intensity.

## Study 2

Since Study 1 showed, as expected, only small changes at the end of the physical anger intensity continuum, we did not collect data for morphs at 10, 20, 80, and 90% intensity. Participants rated the intensity of perceived emotions on an emotion profile, including sadness and anger. They further rated several traits and abilities, which were combined into three scales: Dominance, affiliation and leadership ability.

Dominance and affiliation were selected because they represent central dimensions of interpersonal perception and have been shown to be shaped by emotional expressions^4,5^. Leadership ability was included as a broader, more applied evaluative outcome that has been linked to anger and sadness expressions^31^.

The main questions were: (a) how physical anger intensity was translated into perceived anger and sadness, whether these associations were linear or non-linear, and whether they differed by expresser gender; (b) whether physical anger intensity predicted social judgments of dominance, affiliation, and leadership ability; (c) whether these social judgments were better explained by perceived anger and sadness than by physical anger intensity alone; and (d) whether the associations between perceived emotions and social judgments were linear or non-linear and differed by expresser gender.

### Results

#### Data analysis considerations

To capture the expected non-linear relationship between physical anger intensity and emotion perception we employed Bayesian Spline Modeling (Generalized Additive Models) using the brms package^32^ in R^30^. We estimated distinct, flexible curves for male and female expressers using by-group spline interactions. Non-linearity was evaluated from the posterior distributions of the smooth-term standard deviations (sds): values credibly above zero indicate that the function deviates from a simple linear association. It should be noted that at small values relationships are not necessarily visibly curved.

For dominance, affiliation and leadership ability, we assessed whether perceived emotions and expresser gender predicted the judgment in question. We modeled these variables as a function of gender and two separate thin-plate regression splines for perceived sadness and perceived anger respectively.

All models were estimated using Markov Chain Monte Carlo (MCMC) sampling with four chains and 2000 iterations each (1000 warmup). We used weakly informative priors to allow the data to determine the specific geometry of the splines. Convergence was confirmed via R-hat values (= 1.00), and the adapt_delta parameter was set to 0.95 to ensure stable posterior estimation. For descriptives and model details see supplement (available at OSF).

#### Emotion perception

##### Perceived sadness

Model fit was excellent, $\widehat{R}$ = 1.00, all ESS > 1100, R^2^ = 0.28, residual *σ* = 1.71, CrI_95%_ = [1.60, 1.82]. A reliable negative relationship between physical anger intensity and perceived sadness emerged, such that expressions with a higher percentage of physical anger are rated as less sad. Further, a reliable overall effect of expresser gender, *β =0.24*, CrI_95%_ [0.08, 0.39] emerged, such that male expressers were rated less sad than female expressers at lower anger/higher sadness intensities but sadder at 100% anger intensity. 

**Fig. 3:**
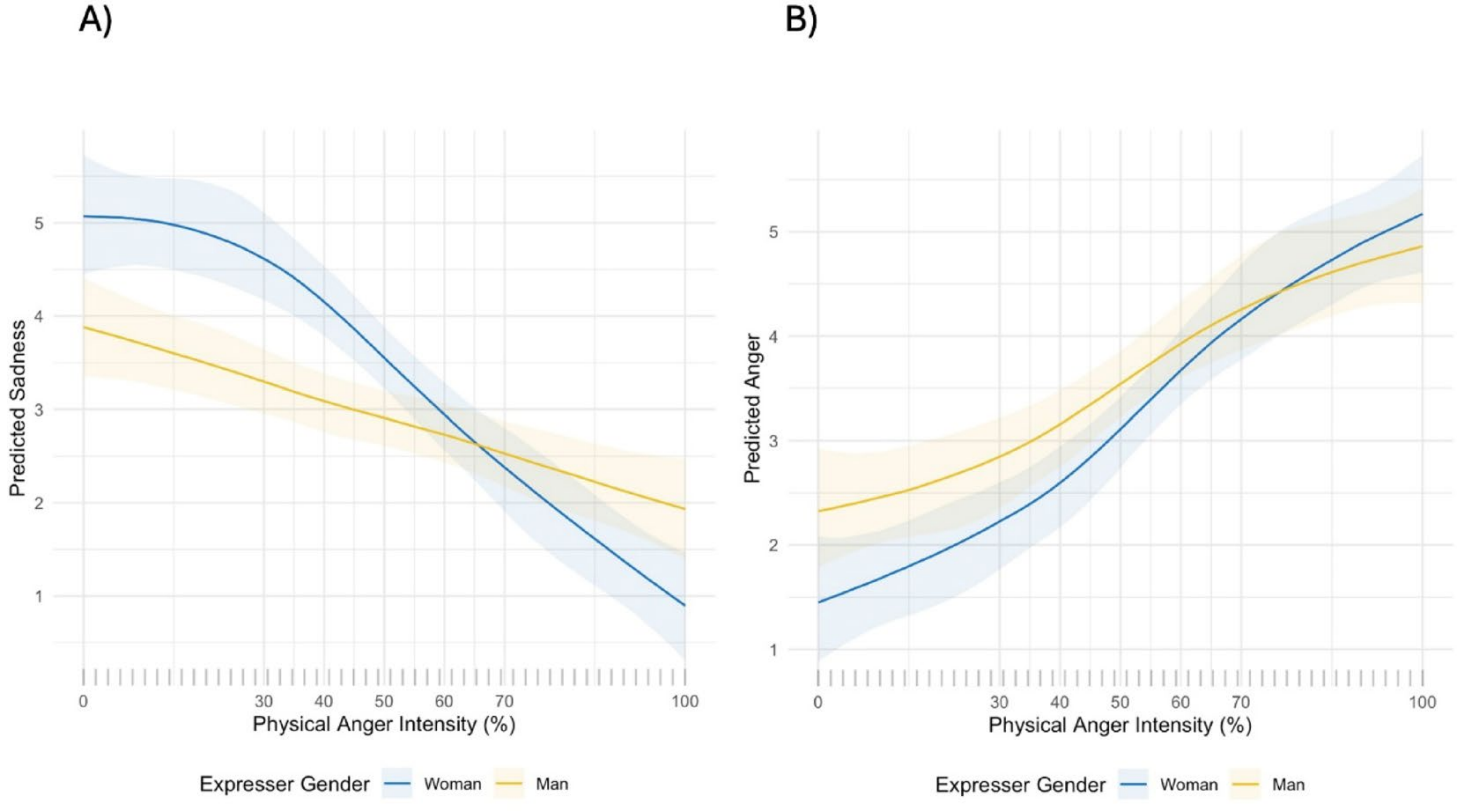
Predicted (**A**) perceived sadness and (**B**) perceived anger as a function of physical anger intensity and expresser gender (for slope estimates and CrIs, see supplement Tables 4 and 5, available at OSF). A value of 0% physical anger corresponds to 100% sadness, whereas 100% physical anger corresponds to 0% sadness.

Reliable sds indicated that the association between physical anger intensity and perceived sadness was non-linear for both female expressers, sds = 1.69, CrI_95%_ [0.27, 4.57], and male expressers, sds = 0.73, CrI_95%_ [0.02, 3.11], with stronger curvature for female expressers. The overall slope was also reliably steeper for female expressers, slope = − 0.047, CrI_95%_ [− 0.055, − 0.039], than for male expressers, slope = − 0.019, CrI_95%_ [− 0.027, − 0.012], Δslope = − 0.027, CrI_95%_ [− 0.038, − 0.017]. Thus, a given increase in physical anger intensity reduced perceived sadness more strongly for female than for male expressers. As shown in Fig. 3A, for female expressers, a given increase in physical anger intensity reduced perceived sadness less at low levels of physical anger, more in the midrange, and then again less for 100% anger. By contrast, for male expressers slopes were largely the same across the continuum, ranging approximately from − 0.021 to − 0.018, approaching linearity.

##### Perceived anger

Model fit was excellent, $\widehat{R}=1.00$, all ESS > *1100*, ${R}^{2}=0.27$, residual *σ =1.68,* CrI_95%_ [1.58, 1.79]. A reliable positive relationship between physical anger intensity and perceived anger emerged, such that expressions with a higher percentage of physical anger were rated as angrier. Further, a reliable overall effect of expresser gender, *β =-0.18*, CrI_95%_ [− 0.34, − 0.02], emerged, such that male expressers were rated angrier than female expressers, when physical anger intensity was low; the difference became smaller for higher physical anger intensities and then (non-reliably) reversed at 100% anger intensity. 

Reliable sds indicated that the association between physical anger intensity and perceived anger was non-linear for both female, sds = 1.36, CrI_95%_ [0.08, 3.98], and male expressers, sds = 1.21, CrI_95%_ [0.03, 3.74]. The overall slope was reliably steeper for female expressers, slope = 0.041, CrI_95%_ [0.033, 0.049], than for male expressers, slope = 0.028, CrI_95%_ [0.020, 0.036], Δslope = 0.013, CrI_95%_ [0.002, 0.024]. Thus, a given increase in physical anger intensity increased perceived anger more strongly for female than for male expressers. As shown in Fig. 3B, for both genders the same increase in physical anger intensity increased perceived anger more in the midrange than at the extremes of physical anger intensity.

In sum, perceived sadness and anger were non-linearly related to physical anger intensity. For anger ratings of both male and female expressers and sadness ratings of female expressers, a given increase in physical anger intensity had stronger effects in the midrange than at the extremes, matching the categorical perception pattern observed in Study 1. Only sadness ratings for male expressers approached linearity.

#### Predicting trait perceptions from physical anger intensity, perceived sadness, perceived anger and expresser gender

##### Dominance

Physical anger intensity reliably predicted perceived dominance, R^2^ = 0.14, CrI_95%_ [0.09, 0.20], such that higher physical anger intensity was associated with higher perceived dominance. This association was non-linear for both female, sds = 0.97, CrI_95%_ [0.02, 3.37], and male expressers, sds = 0.60, CrI_95%_ [0.02, 2.30]. There was no reliable overall effect of expresser gender, β = − 0.09, CrI_95%_ [− 0.23, 0.04].

Perceived anger was positively and perceived sadness negatively related to perceived dominance, matching findings that individuals showing anger are perceived as more dominant than those showing sadness^4,5^. The model had excellent fit, $\widehat{R}=1.00-1.01$, all ESS > *1500*, ${R}^{2}=0.45$, residual *σ =1.17,* CrI_95%_ [1.09, 1.24] and explained substantially more variance in perceived dominance than the model based on physical anger intensity alone.

**Fig. 4:**
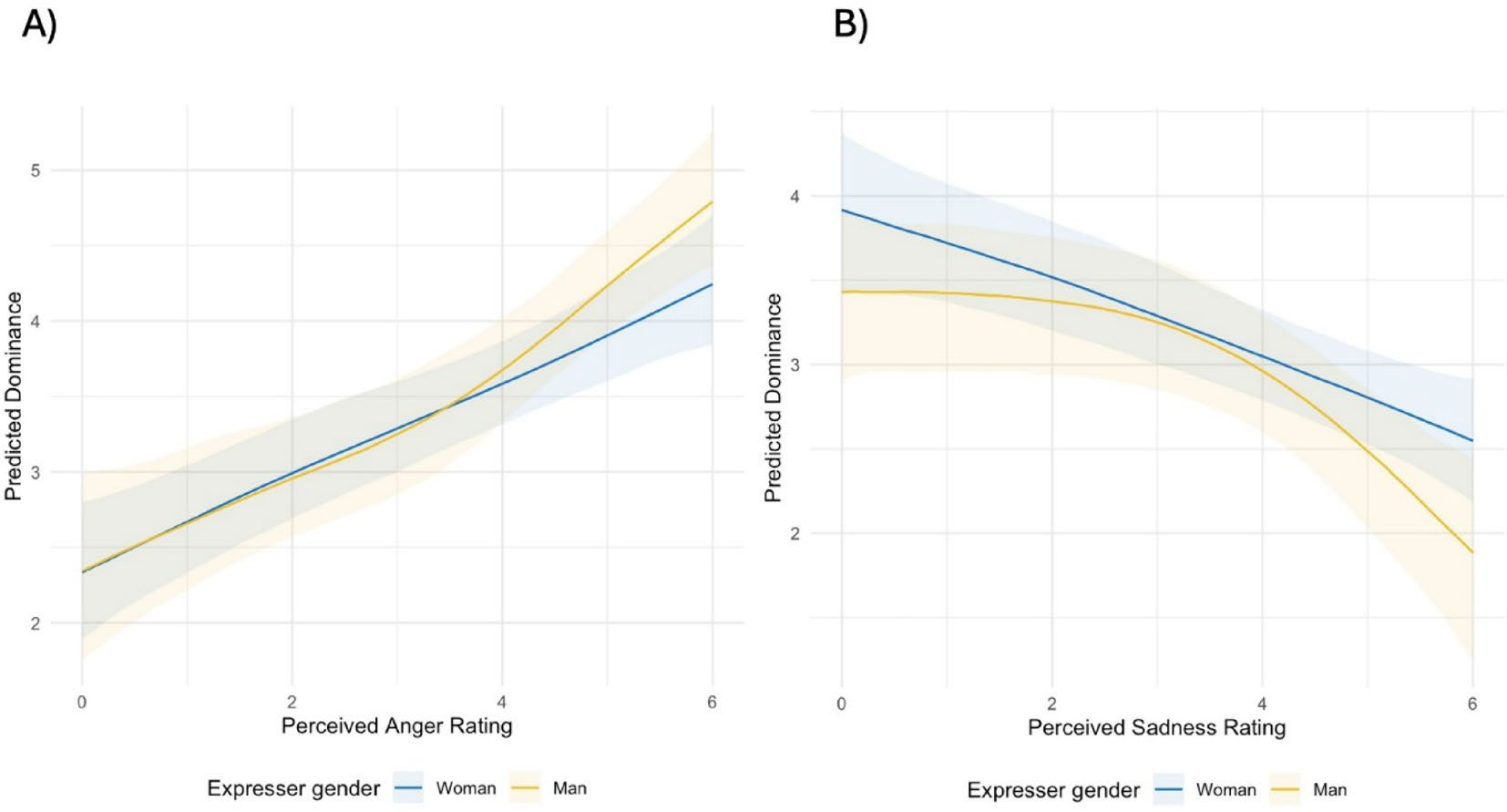
Predicted perceived dominance as a function of perceived anger (**A**) and perceived sadness (**B**) and expresser gender (for slope estimates and CrIs see supplement Table 8, available at OSF).

The overall slopes for female and male expressers did not differ reliably, Δslope_sadness_ = 0.028**,** CrI_95%_ [− 0.128, 0.199]; Δslope_anger_ = − 0.123, CrI_95%_ [− 0.295, 0.034]. The sds indicated reliable non-linear associations for both perceived sadness and perceived anger with smaller sds for female, sds_sadness_ = 0.57, CrI_95%_ [0.02, 2.14]; sds_anger_ = 0.59, CrI_95%_ [0.02, 2.21], than for male expressers, sds_sadness_ = 1.23, CrI_95%_ [0.24, 3.31]; sds_anger_ = 0.99, CrI_95%_ [0.07, 3.15]. As shown in Fig. 4, dominance perceptions for male expressers were less affected by increases in perceived emotions at the lower end of the respective scale but substantially affected by the same increases at the upper end of the scale. By contrast, for female expressers slopes varied very little across the continuum and thus changes in perceived dominance were almost linearly related to perceived sadness and anger (for more details, see the supplement, available at OSF).

##### Affiliation

Physical anger intensity predicted perceived affiliation, R^2^ = 0.11, CrI_95%_ [0.06, 0.16], such that higher physical anger intensity was generally associated with lower perceived affiliation and this association was non-linear for both female, sds = 0.67, CrI_95%_ [0.02, 2.63], and male expressers, sds = 0.46, CrI_95%_ [0.01, 1.83]. There was a reliable overall effect of expresser gender, β = 0.12, CrI_95%_ [0.02, 0.22], indicating higher perceived affiliation for female than for male expressers.

Perceived anger was strongly negatively, and perceived sadness weakly positively related to perceived affiliation but no reliable overall effect of expresser gender emerged, ß = 0.06, CrI_95%_ [− 0.03, 0.15], suggesting that gender differences in perceived affiliation were largely accounted for by differences in perceived anger and sadness. Once perceived emotion was included in the model, the overall gender effect disappeared. The model had excellent fit, $\widehat{R}=1.00$, all ESS > *1100*, ${R}^{2}=0.21$, residual *σ =1.02,* CrI_95%_ [0.95, 1.09] and explained more variance in perceived affiliation than the model based on physical anger intensity alone.

**Fig. 5:**
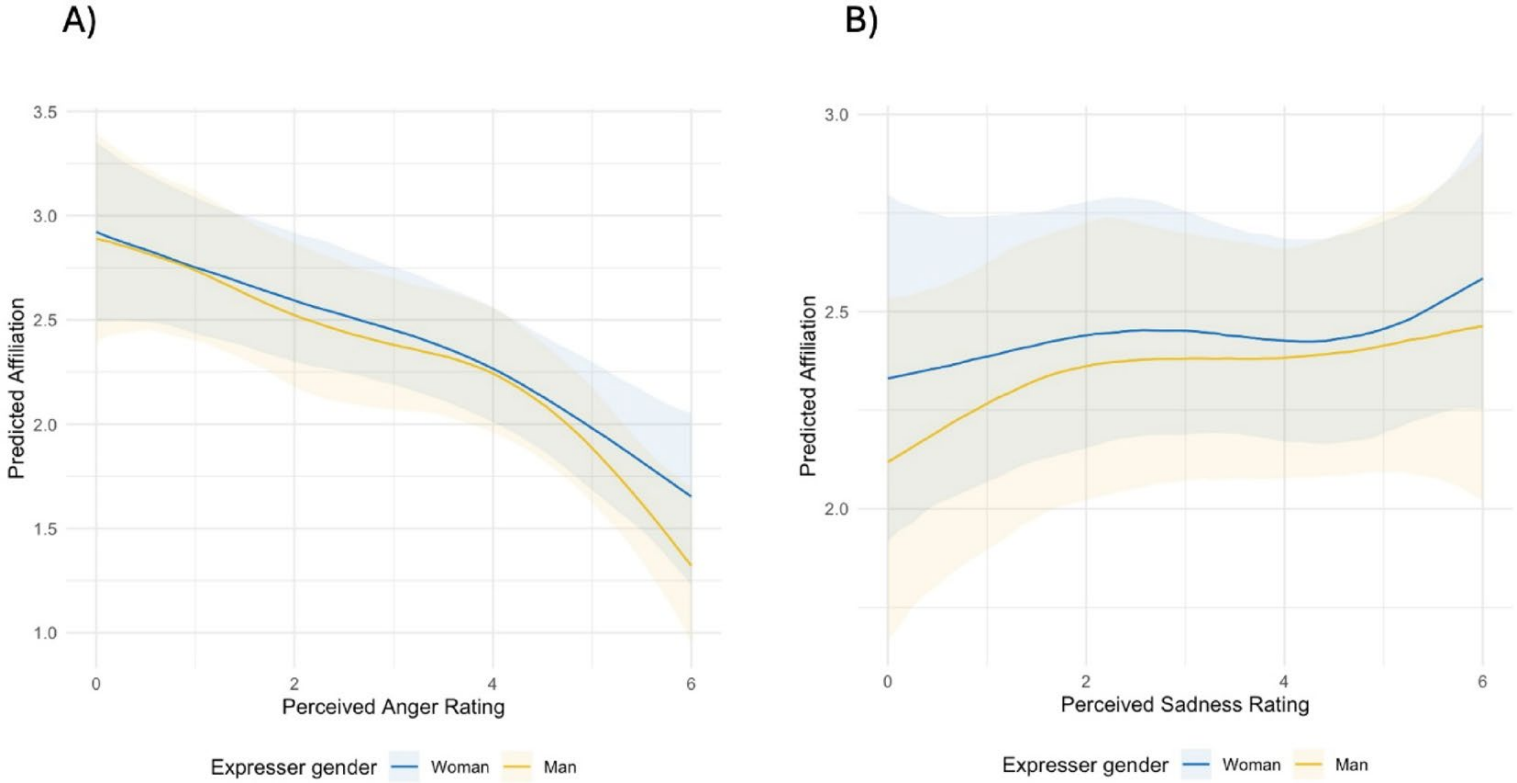
Predicted perceived affiliation as a function of perceived anger (**A**) and sadness (**B**) and expresser gender (for slope estimates and CrIs see supplement Table 11, available at OSF).

For both predictors, the overall slopes for male and female expressers did not differ reliably, Δslope_sadness_ = − 0.011, CrI_95%_ [− 0.148, 0.134]; Δslope_anger_ = 0.104, CrI_95%_ [− 0.057, 0.264]. The sds indicated reliable non-linear associations for both perceived emotions and both expresser genders. For sadness, non-linearity was similar for female, sds_sadness_ = 0.71, CrI_95%_ [0.02, 2.51], and male expressers, sds_sadness_ = 0.67, CrI_95%_ [0.02, 2.47]. For anger, non-linearity was weaker for female expressers, sds_anger_ = 0.71, CrI_95%_ [0.03, 2.42], than for male expressers, sds_anger_ = 1.21, CrI_95%_ [0.11, 3.47]. As shown in Fig. 5, affiliation judgments were shaped mainly by perceived anger: higher perceived anger was associated with lower perceived affiliation for both female and male expressers, with the decrease appearing somewhat stronger for male expressers at higher anger levels. Perceived sadness had overall only small effects on affiliation judgments (for more details, see the supplement, available at OSF).

##### Leadership ability

Physical anger intensity predicted perceived leadership ability only weakly, as the model explained very little variance, R^2^ = 0.05, CrI_95%_ [0.02, 0.09]. The association was non-linear for both female, sds = 0.60, CrI_95%_ [0.01, 2.58], and male expressers, sds = 1.25, CrI_95%_ [0.06, 4.17] and there was no reliable overall effect of expresser gender, β = 0.04, CrI_95%_ [− 0.08, 0.15].

Perceived sadness was negatively associated with perceived leadership ability for both male and female expressers (slope differences were not reliable, Δslope_sadness_ = − 0.024, CrI_95%_ [− 0.200, 0.156]). Perceived anger showed a more gender-specific pattern: Even though slope differences between expresser-gender conditions were not reliable, Δslope_anger_ = − 0.140, CrI_95%_ [− 0.316, 0.042], higher perceived anger was associated with higher perceived leadership ability for male, slope_anger_ = 0.232, CrI_95%_ [0.110, 0.363] but not for female expressers, slope_anger_ = 0.093, CrI_95%_ [− 0.023, 0.221]. No reliable overall effect of expresser gender, ß = 0.11, CrI_95%_ [− 0.01, 0.23], emerged. The model has good fit, $\widehat{R}=1.00-1.01$, all ESS > *500*, ${R}^{2}=0.16$, residual *σ =1.24,* CrI_95%_ [1.16, 1.33] and explained more variance in perceived leadership ability than the model based on physical anger intensity alone.

**Fig. 6:**
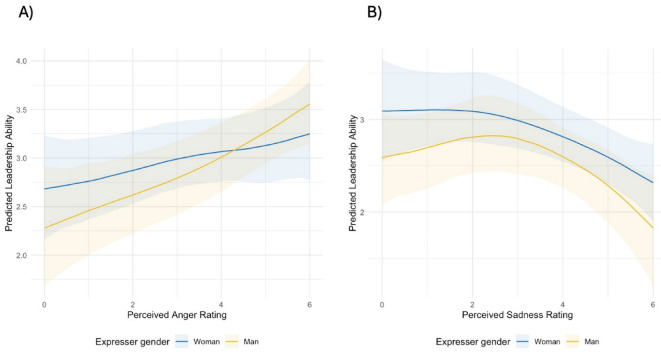
Predicted perceived leadership ability as a function of perceived anger (**A**) and sadness (**B**) and expresser gender (for slope estimates and CrIs, see supplement Table 14, available at OSF).

The effects of perceived sadness and anger on perceived leadership ability were reliably non-linear for both expresser-genders. For perceived sadness, non-linearity was weaker for female, sds_sadness_ = 0.88, CrI_95%_ [0.05, 2.92], than for male expressers, sds_sadness_ = 1.29, CrI_95%_ [0.20, 3.57]. For perceived anger, non-linearity was similar for female, sds_anger_ = 0.68, CrI_95%_ [0.02, 2.53], and male expressers, sds_anger_ = 0.70, CrI_95%_ [0.02, 2.50]. As shown in Fig. 6, the effect of perceived anger was stronger for male expressers at higher perceived anger, and the effect of perceived sadness emerged for both expresser-genders mainly at higher sadness ratings. Interestingly, male expressers were rated as somewhat higher in leadership ability at moderate than at low sadness levels, whereas this pattern was not observed for female expressers. Thus, leadership judgments were more strongly related to perceived emotional meaning than to physical anger intensity alone, which explained only little variance (for more details, see the supplement, available at OSF).

### Discussion

In sum, both emotion perception and social judgments were non-linearly related to physical anger intensity and perceived emotions respectively and the strengths of these relationships differed as a function of expresser gender. Importantly, gender effects were clearest at the level of emotion perception and were substantially reduced once perceived anger and sadness were used to directly predict social judgments. This suggests that expresser-gender differences in person perception were closely tied to differences in the emotional meaning observers assigned to blended expressions, rather than reflecting only direct stereotype-congruent trait attributions.

## General discussion

The present research examined the perception of blended anger–sadness expressions. Across two studies, the findings support three main conclusions. First, Study 1 showed that, in line with prior studies^23,24^, blended anger–sadness expressions were perceived categorically. Specifically, changes at the ends of the continuum had relatively little impact on categorization, whereas changes in the midrange had a much larger effect. Response latencies converged with this interpretation, as categorizations were fastest when one emotion dominated the blend. Second, the model-based category threshold in Study 1 was shifted toward lower physical anger intensities for male relative to female expressers and latency data likewise suggested easier decoding of anger in male faces as anger intensity increased.

In Study 2 as well, perceptions of sadness and anger varied non-linearly across the physical anger continuum and gender differences emerged. Female faces were rated as sadder than male faces at lower anger/higher sadness levels, whereas male faces were rated as angrier at lower and moderate anger intensities; this difference attenuated and eventually reversed at the highest anger intensity. Thus, the more familiar gendered pattern was clearest at lower and intermediate physical anger intensities rather than at the extreme anger endpoint. The reversal at higher physical anger intensities may reflect a shifting-standards effect^33^. Because anger is less congruent for female expressers and sadness less congruent for male expressers, strongly anger- or sadness-looking faces may have been perceived as especially diagnostic when they violated gendered expectations.

Importantly, our findings suggest that conclusions based on highly prototypical endpoint stimuli do not generalize straightforwardly across the full continuum of mixed expressions. At the same time, they remain consistent with the idea that anger is more salient, expected or both when displayed by men^25,26^.

Third, non-linearity carried over to social judgments, but the precise form of the relationship depended on the judgment in question. Social impressions from mixed expressions were not determined by physical signal strength alone, but by a more complex interplay of physical intensity, perceived emotion and expresser gender.

Importantly, gendered effects on social judgments were largely determined by biases in emotion perception. Observers perceived different levels of sadness and anger in the same blends shown by male and female expressers but then attributed very similar trait levels based on a given perceived emotion intensity. In this sense, the present findings are compatible with reverse-appraisal accounts of trait inference from emotion expressions^11,12^: Observers appear to use perceived emotional meaning as a basis for inferring broader characteristics, such as dominance, affiliation and leadership ability, rather than basing these attributions predominantly on stereotype expectations.

Broadly consistent with this interpretation, perceived anger predicted higher dominance and perceived sadness predicted lower dominance for both expresser-gender conditions. Thus, dominance judgments appear to track the emotional meaning of the face as expected: anger signals agency and control, whereas sadness signals reduced perceived coping potential. At the same time, the stronger non-linearity for male expressers in the rated-emotion model suggests that once observers perceive anger or sadness in male faces, changes at the upper end of these perceived emotion scales may carry disproportionate weight for dominance judgments.

A somewhat different pattern emerged for affiliation. As physical anger intensity increased, faces were judged as less affiliative overall and female expressers were judged as more affiliative than male expressers across the continuum. Yet this gender effect disappeared once perceived sadness and anger were entered directly into the model. For both genders only perceived anger was strongly associated with lower affiliation whereas perceived sadness showed only weak and unreliable positive associations. This pattern is especially informative because it suggests that the female advantage in perceived affiliation was largely accounted for by the fact that female faces were perceived as less angry at comparable physical intensity levels. In other words, gender seems to shape affiliation judgments less through a direct trait stereotype effect than through its effect on the emotional meaning observers assign to the face.

Leadership judgments showed still another pattern. Physical anger intensity alone explained little variance in perceived leadership ability. Yet, when perceived emotions were entered directly, a clearer pattern emerged. Perceived anger was positively related to leadership ability but only for male expressers, whereas perceived sadness was negatively related to leadership ability for both genders. Thus, as with dominance, leadership judgments appear to depend on the extent to which the face conveys agentic rather than low-coping emotional meaning. In fact, for female expressers perceived leadership ability depended only on the degree to which they did not appear sad.

In sum, the present findings only partially matched the stereotype-based expectations outlined in the introduction. Although female expressers were judged as more affiliative overall and male faces were categorized as angry at lower physical anger intensities, the consequences of perceived anger and sadness for dominance and affiliation judgments were not simply stereotype-consistent. Instead, the results suggest that stereotype-related gender effects operate primarily by shaping how the same physical signal was emotionally interpreted across the continuum, rather than by exerting a simple additive effect on trait attribution.

A broader implication of the present work is that linear models may obscure important subtleties for person perception from facial expressions. The present analyses show that across emotion ratings and person judgments, several of the observed functions were clearly non-linear, although the degree and form of non-linearity varied by outcome and gender.

This matters because a linear account assumes that each incremental increase in anger intensity contributes equally to judgment. By contrast, the present findings point to threshold regions, plateaus, and gender-specific changes in slope. Put differently, the psychological meaning of moving from 40 to 70% anger is not necessarily the same as moving from 70 to 100% anger. This is especially important because much previous work on trait attribution from facial expressions has relied on highly intense, prototypical expressions^4–7^). By contrast, spontaneous emotion expressions are generally much weaker^34^ as well as more likely to be blended. As such, the non-linear effects observed in the midrange of the continuum may be especially relevant for understanding everyday expressions, which are often weaker and more ambiguous.

Taken together, our findings suggest that social judgments of blended facial expressions are not well captured by simple linear assumptions. Study 1 showed that anger–sadness blends are perceived categorically and that male faces are categorized as angry at lower physical anger intensities than female faces. Study 2 further showed that this perceptual non-linearity carries over to person judgments, but not uniformly across outcomes. At the same time, the role of expresser gender depended on the judgment in question. Gender differences were especially evident at the level of emotion perception but were reduced once rated emotions were entered directly. This pattern suggests that gender shapes social judgments largely through its influence on how emotional intensity is perceived in the first place.

### Strengths and limitations

Two well-powered studies provided novel insights into the relationship between social perceptions and physical emotion intensity in blended emotion expressions. Using Bayesian spline models allowed us to model non-linear functions flexibly despite the uneven spacing of the intensity levels.

Yet several limitations should also be noted. First, the stimuli were static, morphed facial images derived from a limited set of identities from the young adult subset of the FACES database^28^. Although the use of a standardized stimulus set provided a high degree of experimental control and reduced the likelihood of uncontrolled differences between male and female expressers, the expressions were decontextualized and highly constrained. Moreover, the stimuli were not intended to be representative of everyday interactions, where mixed emotions are typically embedded in dynamic facial movement and accompanied by vocal cues, bodily behavior, and situational context.

Second, the present work focused on only one emotion blend, namely anger and sadness. This blend was chosen because anger and sadness differ in appraisal implications related to agency, dominance, and coping potential, all relevant for our focal social judgments. Research on the perception of emotion blends^23^ demonstrates that all studied blends were perceived categorically, thus similar non-linearity should emerge for other blends, but this needs to be verified in future research.

Also, both studies relied on online presentation. Thus, viewing conditions such as screen size, distance, and lighting could not be controlled as precisely as in a laboratory setting and this may have added noise to the data. However, participants were restricted to using a computer rather than a smartphone or tablet, which reduced some of this variability.

## Conclusion

Overall, the present findings show that blended anger–sadness expressions are interpreted in ways that are both gendered and non-linear. Observers do not simply read physical intensity off the face in a one-to-one fashion. Instead, the same physical signal is transformed by categorical emotion perception and by gendered expectations, and these processes in turn shape judgments of dominance, affiliation and leadership. More broadly, the findings suggest that understanding social perception from facial expressions requires moving beyond simple linear assumptions and taking seriously the possibility that small changes in emotional intensity can have qualitatively different social meanings depending on where they occur on the continuum and who is expressing them.

## Methods

The studies were approved by the Ethics Committee of The Herta & Paul Amir Faculty of Social Sciences, University of Haifa (No. 362/25). All procedures were carried out in accordance with relevant institutional guidelines and regulations and with the Declaration of Helsinki. Informed consent was obtained from all participants prior to participation.

### Methods: study 1

#### Participants

Ninety-two participants (75 women, 14 men, 3 other) with a mean age of 26 years (SD = 7.5) were recruited at the Department of Psychology at Humboldt University of Berlin and received course credit. Data from one participant were excluded because they failed to respond within 2000 ms on more than 50% of the trials.

##### Power considerations

Previous studies focusing on categorical perception (e.g.^23,24^) used very small samples relying on large trial numbers instead. However, these studies did not examine whether the category boundary, that is, the point at which expressions are equally likely to be categorized as one emotion or the other (point of subjective equality; PSE), shifts as a function of expresser gender. Hence, we aimed for a larger sample by collecting data until the end of the term.

To assess the sensitivity of our design to an expresser gender difference in PSE, we conducted a simulation-based sensitivity analysis using simr^35^ in R^30^ based on the final sample size (N = 91). Anger intensity was centered at 50%. We modeled bias from 2 to 8% by varying the main effect of expresser gender relative to the observed psychometric slope (*β =6.37)*. Results indicated that our design achieved 80% power for a shift of 5%.

#### Stimuli

The total stimulus pool consisted of 220 expressions representing ten male and ten female identities, selected from the young adult subset of the FACES database^28^. For each identity, an 11-step morph continuum was created using FantaMorph^36^ ranging from 100% sadness/0% anger to 0% sadness/100% anger in 10% increments.

The continua were standardized in terms of their physical composition, such that each morph level contained the same physical proportion of sadness and anger across expresser genders. However, as the expressions were taken from a well-validated face data base, the endpoints were not additionally pretested.

Given that the full design with 220 stimuli would have been too demanding for participants, a between-subjects design was employed for expresser gender. Participants were randomly assigned to view either 110 male stimuli or 110 female stimuli.

#### Procedure

The experiment was programmed and administered online using Inquisit software (Version 5.0; Millisecond Software). After providing informed consent and receiving standardized instructions to respond as quickly and accurately as possible, participants performed a two-alternative forced-choice task to categorize each expression as “Anger” or “Sadness”. For each trial, a facial expression was presented simultaneously with two emotion category buttons. The stimulus remained on screen for 2000 ms or until a response was registered. The right/left position of the “Anger” and “Sadness” buttons was counterbalanced between participants. Following the experimental block, participants provided demographic information. The dependent variables were categorization choice and response latency for each of the 11 morph levels. Trials for which latencies exceeded the maximal presentation time (2000 ms) were excluded. Participants then completed an emotion rating task, which is not part of the present report.

### Methods: study 2

#### Participants

Complete data from 449 participants were collected. Data from 2 participants were excluded because they failed both attention checks. The final sample consisted of 447 participants (225 men, 220 women, 2 other) with a mean age of 44.1 years (SD = 12.6) who were recruited via Prolific. Participants were paid £ 0.75 for the 3-min task.

#### Stimuli

We retained the 0, 30, 40, 50, 60, 70, and 100% anger intensity morphs from 4 male and 4 female identities, selected from the young adult subset of the FACES database^28^, which were used in Study 1.

The study used a complete between-subjects design: each participant rated one stimulus only. We used this design to reduce demand and anchoring effects when participants make complex social judgments on only subtly different emotion blends. The design comprised two expresser genders and the seven physical anger intensity levels (see above) with the four identities nested within each expresser-gender condition. To ensure adequate cell sizes, we aimed for N ≥ 30 per expresser gender x physical anger intensity cell^37^.

#### Dependent variables

Participants saw one face and rated the degree to which the expresser seemed competent, dominant, submissive, sociable, friendly, warm, kind, and cold. In addition, they rated the extent to which the expresser seemed able to get others to do what they want and to have control over the situation. The trait ratings were combined into three scales: dominance, affiliation, and leadership ability (see below). Dominance and affiliation were included because they map directly onto core interpersonal dimensions and have been shown to be shaped by emotional expressions such as anger and sadness^4^. The two additional items were included to capture a broader, more applied judgment of social influence and perceived control.

Finally, participants rated the degree to which they perceived the expresser to express: Sadness, Anger, Disgust, Fear, Happiness, Shame, and Neutrality (no emotion). All ratings were made on scales anchored with 0 = *not at all* and 6 = *to a large extent*. Because the morph continuum varied only anger and sadness, the analyses focused on perceived anger and perceived sadness; the remaining ratings were included as part of the broader emotion profile but were not central to the present research questions.

We conducted a principal components analysis using the psych package^38^ with a Varimax rotation. Visualization of eigenvalues was generated via factoextra^39^. A two-factor solution that explained 78% of the variance was chosen based on the scree-plot (see supplement for details, available at OSF). Due to high cross loadings (> 0.5) competent was excluded from further analysis. Based on this analysis, two composite scores were created: dominance (dominant and reverse-scored submission, *α* = 0.85) and affiliation (sociable, friendly, kind, warm and reverse-scored cold, *α* = 0.91). The two leadership items were highly correlated, r = 0.70, and were combined into a single leadership ability scale, α = 0.84.

#### Procedure

The experiment was programmed and administered online using JATOS^40^. After informed consent and standardized instructions, participants saw one face and were asked to rate the expression with regard to the expresser’s traits and emotion expression. Following the experimental block, participants provided demographic information, were debriefed, and received a code for payment.

## Data Availability

All data and complete markdowns are available at OSF.
